# Electroacupuncture regulates histone acetylation of Bcl-2 and Caspase-3 genes to improve ischemic stroke injury

**DOI:** 10.1016/j.heliyon.2024.e27045

**Published:** 2024-03-04

**Authors:** Lingling Meng, Bufan Wu, Ling OuYang, Rou Peng, Yonglin Chen, Zhijuan Tang, Min Zhang, Tanqing Xu, Yaling Wang, Shengfeng Lu, Xinyue Jing, Shuping Fu

**Affiliations:** Key Laboratory of Acupuncture and Medicine Research of Ministry of Education, Nanjing University of Chinese Medicine, Nanjing, 210023, China

**Keywords:** Histone acetylation, Electroacupuncture, Cerebral ischemia-reperfusion, Apoptosis, Epigenetics

## Abstract

**Background:**

Imbalances between Bcl-2 and caspase-3 are significant evidence of apoptosis, which is considered an influential factor in rapidly occurring neuronal cell death and the decline of neurological function after stroke. Studies have shown that acupuncture can reduce poststroke brain cell damage via either an increase in Bcl-2 or a reduction in caspase-3 exposure. The current study aimed to investigate whether acupuncture could modulate Bcl-2 and caspase-3 expression through histone acetylation modifications, which could potentially serve as a neuroprotective mechanism.

**Methods:**

This study used TTC staining, Nissl staining, Clark neurological system score, and Evans Blue (EB) extravasation to evaluate neurological damage following stroke. The expression of Bcl-2/caspase-3 mRNA was detected by real-time fluorescence quantification of PCR (real-time PCR), whereas the protein expression levels of Bcl-2, Bax, caspase-3, and cleaved caspase-3 were assessed using western blotting. TUNEL staining of the ischemic cortical neurons determined apoptosis in the ischemic cortex. Histone acetyltransferase (HAT) and histone deacetylase (HDAC) activities, along with the protein performance of AceH3, H3K9ace, and H3K27ace, were detected to evaluate the degree of histone acetylation. The acetylation enrichment levels of H3K9 and K3K27 in the Bcl-2/caspase-3 gene were assessed using Chromatin Immunoprecipitation (ChIP) assay.

**Results:**

Our data demonstrated that electroacupuncture (EA) exerts a significant neuroprotective effect in middle cerebral artery occlusion (MCAO) rats, as evidenced by a reduction in infarct volume, neuronal damage, Blood-Brain Barrier (BBB) disruption, and decreased apoptosis of ischemic cortical neurons. EA treatment can promote the mRNA and protein expression of the Bcl-2 gene in the ischemic brain while reducing the mRNA and protein expression levels of caspase-3 and effectively decreasing the protein expression levels of Bax and cleaved caspase-3. More importantly, EA treatment enhanced the level of histone acetylation, including Ace-H3, H3K9ace, and H3K27ace, significantly enhanced the occupancy of H3K9ace/H3K27ace at the Bcl-2 promoter, and reduced the enrichment of H3K9ace and H3K27ace at the caspase-3 promoter. However, the Histone Acetyltransferase inhibitor (HATi) treatment reversed these effects.

**Conclusions:**

Our data demonstrated that EA mediated the expression levels of Bcl-2 and caspase-3 in MCAO rats by regulating the occupancy of acetylated H3K9/H3K27 at the promoters of these two genes, thus exerting a cerebral protective effect in ischemic reperfusion (I/R) injury.

## 1 Introduction

Stroke is an acute neurological disorder syndrome caused by localized blood circulation disorders in the brain and is characterized by high morbidity, disability, and mortality [[1], [2], [3], [4]]. Ischemic stroke is one of the most common types of stroke, with more than 85% of patients experiencing cerebral ischemia due to a rapid reduction in blood supply [5]. At the center of the infarct, cell death is primarily due to necrosis caused by irreversible neuronal damage [6,7]. Apoptosis is the primary mode of cell death in the penumbra surrounding the ischemic focus, a hypoperfused area that has not yet been infarcted [8,9]. Apoptosis is the process of scheduled cell death that can be triggered by various stimuli and is one of the most typical and prevalent forms of controlled cell death [10]. Both Bcl-2 family proteins and the caspase family play critical roles in apoptosis activation, signaling, and execution [11]. Bcl-2 is a critical regulatory factor of the Bcl-2 family and is known for its anti-apoptotic role. In contrast, Bax is a pro-apoptotic protein belonging to the Bcl-2 family. The activation of Bax leads to an increase in mitochondrial outer membrane permeability, releasing pro-apoptotic factors into the cytoplasm and initiating a series of apoptotic signaling cascades within the cell. Caspase-3 is the predominant protease involved in apoptotic signaling pathways. The activated form of caspase-3, cleaved caspase-3, is a crucial executioner caspase in the apoptotic pathway, and is often considered a hallmark of apoptosis [12]. Previous studies have shown that 24 h after ischemic stroke, both apoptotic caspase-3 and anti-apoptotic Bcl-2 are simultaneously overexpressed in the ischemic area [13]. Because damage to the penumbral zone may be reversible [14], identifying strategies to prevent apoptosis is vital and helps mitigate the death of peripheral neurons after stroke.

Electroacupuncture (EA), which originated from traditional Chinese medicine and is now combined with modern electrical stimulation therapy, is a commonly used treatment for various clinical conditions, including ischemic stroke [[15], [16], [17]]. Studies have suggested that EA alleviates neurological disorders in stroke survivors without causing significant side effects [18]. Experimental studies have confirmed that EA reduces infarct volume by ameliorating neurological disturbances and decreasing the number of ischemic pathologies [[19], [20], [21]]. An increasing number of studies have indicated that the inhibition of neuronal apoptosis is an essential route of action of EA in stroke. Tang et al. found that EA treatment could downregulate the activation of cleaved caspase-3, reduce neuronal apoptosis, and dramatically reduce neurological impairment and the volume of cerebral infarcts in rats with ischemic stroke [22]. Chen et al. identified that EA at the Baihui (GV 20) and Shenting (DU 24) acupoints could significantly inhibit the expression of caspase-3, improve the neuromotor function and cognitive performance of stroke rats, and alleviate cerebral cortical hemisphere injury after cerebral ischemia [23]. Kim et al. confirmed that after EA treatment, the number of apoptotic cells in the brain of rats with ischemic stroke decreased significantly, the expression of Bcl-2 and Bcl-xL increased, and the activity of the caspase-3 enzyme and the expression of cleaved caspase-3 protein decreased [24]. These studies provide strong evidence that EA treatment exerts anti-apoptotic effects by upregulating the expression of Bcl-2 and downregulating the expression of caspase-3 in the cerebral ischemia model of cerebral ischemia. However, the mechanism by which acupuncture regulates the expression of caspase-3 and Bcl-2 requires further investigation.

Histone acetylation is one of the most studied mechanisms of gene transcriptional activity in epigenetics and is mediated by histone acetyltransferases (HAT) and histone deacetylases (HDAC) [25]. It also plays important roles in the process of cellular apoptosis. In a study conducted by Yan et al. histone acetylation was found to regulate H3K9 acetylation near the promoter region of apoptosis-related genes, leading to the downregulation of caspase-3 and caspase-8 expression, thus inhibiting cellular apoptosis [26]. Xu et al. reported that EA increased the enrichment of H4K16ace on the promoter of Beclin1 and alleviated ischemic reperfusion (I/R) injury, suggesting that acupuncture may affect programmed cell death by modulating histone acetylation [20]. Our previous study found that EA treatment promoted VEGF angiogenesis in a model of myocardial ischemia by increasing H3K9ace occupation of the VEGF promoter region, thus upregulating VEGF expression [27]. In addition, EA treatment reduced HDAC activity in rats with cerebral ischemia [28]. These results indicate that the protective effect of EA is associated with histone acetylation.

In this study, we conducted EA treatment at the Baihui (GV20) acupoint in rats with middle cerebral artery occlusion (MCAO) and examined the relationship between Bcl-2/Caspase-3 transcriptional activity and the enrichment of H3K9 ace/H3K27ace to explore whether the protective effects of EA against cerebral ischemic injury are due to modifications of histone acetylation on the promoter of target genes.

## 2 Materials and methods

### 2.1 Animals and laboratory design

**Fig. 1:**
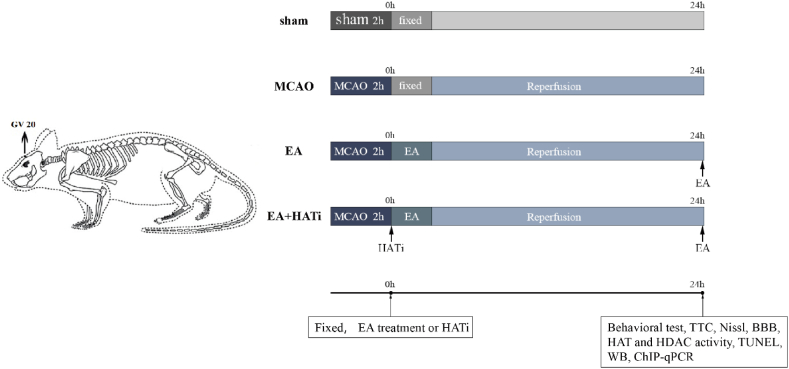
Experimental procedure and grouping. The sham group only uses sham surgery. The MCAO group, the EA group, and the EA + HATi group were reperfused for 1 day after receiving MCAO surgery, thereby establishing a model of cerebral ischemia-reperfusion injury. The rats in the EA group and the EA + HATi group were treated with EA. GV20, also known as Baihui, is an acupuncture point located at the top of the head. It is situated at the intersection of the sagittal midline and the line connecting the apexes of the ears. In order to observe the involvement of histone acetylation in EA treatment, the rats in the EA + HATi group were injected with HAT inhibitor II (4 mg/kg) immediately after the successful model establishment. Clark's neurological function score, TTC staining, Nissl staining, Evans blue, HDAC and HAT activity, TUNEL, Western blotting, ChIP and RT-qPCR were studied 24 h after the injection.

Adult male pathogen-free (SPF) Sprague-Dawley (SD) rats (7–8 weeks old, 250–300 g) from the Nanjing University of Traditional Chinese Medicine Laboratory Animal Center were used in the experiment. Rats (4–5 rats per cage) were placed in a temperature-controlled, light-dark cycled room with an ambient temperature of 25 °C and a humidity of 60%, respectively, and free access to food and water. After three days of adaptive feeding [29], the rats were randomly assigned to four groups: the sham-operated group (sham group), the MCAO group, the MCAO + EA group (EA group), and the MCAO + EA + Histone Acetyltransferase inhibitor II (EA + HATi group) (Fig. 1).

The experimental workflow is illustrated in Fig. 1. All experiments were performed in accordance with the National Institutes of Health Guide for the Care and Use of Laboratory Animals. The Animal Experiment Ethics Board of Nanjing University of Traditional Chinese Medicine granted permission for all procedures (approval number: 201911A018). All surgeries and executions on animals were conducted under isoflurane anesthesia, for which every possible attempt was made to minimize the pain of the animals existed.

### 2.2 MCAO surgery

The MCAO model was established using the modified filament embolization method [30]. Briefly, animals underwent preoperative fasting for 12 h, followed by 5% isoflurane anesthesia, and exposure to the left external cervical artery (ECA), left common carotid artery (CCA), and internal carotid artery (ICA) was performed with a midline incision. Insert a 3-0 silicone wire (0.26 mm diameter) through the ECA stump into the ICA stump to shut off the left middle cerebral artery (MCA). Two hours after the blockage, the nylon cord was gradually removed to allow reperfusion and restoration of oxygen flow in the MCA region. Rats in the sham group underwent a similar procedure but without blockage of the MCA.

### 2.3 Neurobehavioral valuation

Neurological outcomes were assessed using Clark's method [31] with a single-blinded experimental design. Briefly, neurological deficits were assessed using a 28-point scoring system 24 h after I/R. Seven areas were examined: physical body synchrony, stance, climbing, rotational experiment, forelimb symmetry, mandatory rotation, and whisker responsiveness. Each area contains five scoring criteria. A total number of Seven combined test points resulted in individual scores for each animal.

### 2.4 EA treatment

EA stimulation was performed using a Baihui (GV20). After I/R injury, rats in the EA and EA + HATi groups received once-daily EA. The first EA session was performed 2 min after recovery from the cerebral flow. The last therapy session was administered approximately 2 h before execution. Our team's previous experimental studies have confirmed the neuroprotective effect of this EA treatment protocol on the brain [32]. GV20 lies centrally on the line linking the left ear to the tip of the right ear. Needles were inserted subcutaneously into the GV20 point at an approximate distance of 3 mm, and the needles were connected to Han's EA instrument for the EA group. EA stimulation was applied at a current of 1.0 mA and a frequency of 2/15 Hz for 30 min [33]. In contrast, the sham and MCAO groups were immobilized in the same manner but did not receive EA stimulation and were fixed for 30 min only.

### 2.5 Drug intervention

The HATi II (EMD Millipore Corporation, #382110) powder was diluted into a 1 mg/mL storage solution with PBS with a storage capacity at 4 °C. The HATi II solution was injected intraperitoneally within 2 min of cerebral blood flow reperfusion. A 4 mg/kg dose was administered with one injection per rat.

### 2.6 Specimen collection

In the EA group and the EA + HATi group, animals were injected with pentobarbital at an excessive intraperitoneal dose 2 h after the EA treatment was completed before death, and the animals were immediately flushed with 0.9% saline by perfusion through the heart. The brains were quickly removed and placed on ice for subsequent assays after the clear and bright fluid flowed out of the heart ear. The MCAO and sham groups were processed in the same way as described above.

### 2.7 TTC staining

After completing the neurological functional evaluation, the rats that underwent 1-day I/R injury were anesthetized, and their whole brains were rapidly extracted. The morphologically intact brains were immediately placed in a −20 °C freezer for 15 min. Subsequently, 30 mL of 2% TTC staining solution was prepared and evenly poured into a light-shielded six-well plate. The plate was then placed in a preheated 37 °C oven for 10 min. The frozen brain tissue was sliced to ensure complete immersion in the TTC solution. The slices were then incubated in a 37 °C incubator for 20 min, with a flip every 6 min to ensure even staining. Ischemic brain tissue appeared pale. After incubation, tissue slices were fixed with 4% paraformaldehyde and photographed the following day. Finally, the ImageJ software was used for image processing and statistical analysis of the infarct area.

### 2.8 Nissl staining

Rat brain cryosections were 20 μm thick. After being removed from the −20 °C freezer, the frozen sections were allowed to thaw at room temperature for 30 min. Following a wash with PBS, the sections underwent progressive dehydration using a series of graded ethanol solutions (75%, 90%, 100%, 90%, and 75%). Subsequently, the sections were rinsed with distilled water. The sections were then immersed in a Nissl staining solution, and the stained slides were placed in a thermostat set at 60 °C for 25 min. After a brief rinse with distilled water, the sections were rapidly differentiated in 95% ethanol for 1 min. Dehydration was achieved using xylene for 5 min, followed by fixation with a neutral resin. After three days of air-drying in a fume hood, Nissl bodies in the ischemic cortex of rats were observed using light microscopy.

### 2.9 Evans blue (EB) extravasation

EB extravasation assays were performed 1 d after cerebral I/R injury to evaluate Blood-Brain Barrier (BBB) permeability. Briefly, after the rats were tranquillized, an EB solution (2%, 4 ml/kg, Sigma-Aldrich) was injected into the femoral vein, where it was circulated for over 60 min. Phosphate-buffered saline (PBS) was then perfused through the heart to remove blood cells. The ischemic lateral hemisphere was weighed and equalized with 3 mL/600 mg of N, N-dimethylformamide (Sigma-Aldrich). were allowed to culture at 55 °C for 24 h and incubated, followed by centrifugation (13,000 rpm for 20 min). Using a microplate reader, EB dye in the supernatant was measured at 632 nm.

### 2.10 HDAC and HAT activity assays

To measure the activity of both HDAC and HAT, the HDAC Activity Assay Kit (Millipore, item #17–356) and HAT Activity Assay Kit (BioVision, item #K334-100) were used according to the manufacturer's instructions. Proteins were extracted from fresh brain tissue and quantified. HDAC and HAT activity was estimated using 20 μg of protein. Briefly, for the HDAC activity assay, the HDAC assay buffer containing TSAs was used as an inhibitor control, HeLa nuclear extract was used as a positive control, and the HDAC assay buffer solution was used as the negative control. Subsequently, the enzyme plate was placed in an ELISA reader for the assay, with excitation light at 350–380 nm and emission light at 460 nm. The detected optical density value's magnitude reflected the HDAC activity level.

For the determination of the HAT activation, dissolve 20 μg brain tissue proteins in 40 μl of H_2_O (ultimate amount) and then with 5 μl of specific color substrates 1 and 2 based on a manufacturer's directions. 50 μl of 2 × HAT assay buffer and 8 μl of NADH-generating enzyme were added. After thorough mixing, the samples were incubated at 37 °C for 4 h. Subsequently, the samples were placed in an enzyme calibrator for detection at a reading wavelength of 440 nm. The magnitude of the value obtained reflected the level of HAT activity.

### 2.11 Western blotting

Western blotting was used to determine the quantity of target proteins being made in the ischemic hemisphere. Briefly, brain tissues were milled in liquid nitrogen, and proteins were extracted using RIPA cleavage buffer. Protein intensity, with BSA as a standard, was measured according to the Bradford method. Each sample was separated using 12% SDS-PAGE and transferred onto a methanol-activated PVDF membrane (Millipore, Burlington, MA, USA) by wet electrotransfer. The membranes were blocked for 1.5 h at room temperature with 5% BSA. The membranes were then incubated with primary antibodies against Ace-H3 (Millipore, cat #9717, 1:1000), H3K9ace (Cell Signaling Technology, cat #6949, 1:1000), H3K27ace (Cell Signaling Technology, cat #4353, 1:1000), Histone3 (Abcam, ab1791, 1:1000), Bcl-2 (Abcam, ab59348, 1:1000), Bax (Cell Signaling Technology, cat #2772, 1:1000), caspase-3 (Cell Signaling, cat #9662, 1:1000), cleaved caspase-3 (Cell Signaling, cat #9664S, 1:1000) and GAPDH (SAB, cat #21612S; 1:1000). The total amount of ECL liquid was absorbed in a 1:1 ratio (solution A: B) to uniformly cover the entire film and was observed using an AI600 imaging system (GE Healthcare, USA). GAPDH was used as an internal reference for comparison of grayscale values.

### 2.12 TUNEL assay for cell apoptosis detection

Frozen rat brain tissue sections were equilibrated at room temperature for 30 min and fixed in 4% paraformaldehyde for 30 min to preserve cellular structures. Subsequently, the sections were washed with PBS for 5 min to remove excess paraformaldehyde and other contaminants. A balancing solution was applied for 10 min at room temperature to create an optimal environment. Then, a 20 μg/ml proteinase K solution was added and incubated at 37 °C for 30 min. After proteinase K digestion, the sections were washed thrice with PBS for 5 min each. Next, the sections were incubated in a light-protected constant temperature chamber at 37 °C for 60 min with the TUNEL detection solution. Following the TUNEL assay, the sections were washed thrice with PBS for 5 min each to remove unutilized reagents and impurities. For nuclear staining, 10-min incubation with a working solution of DAPI, a fluorescent dye that binds to DNA and labels cell nuclei, was performed. Finally, sections were sealed with an anti-fluorescence fading mounting medium to prevent fluorescence quenching. Fluorescence imaging was performed to observe and document apoptosis.

### 2.13 Real-time (RT)-qPCR

Briefly, the total RNA from the ischemic hemispheric cerebral organization mentioned above by TRIzol® agent (Invitrogen) as per instructions of manufacture. Reverse RNA recording was performed using a retro-transcription kit (Thermo Fisher Scientific). Fluorescence-based PCR was conducted using a Viia7 real-time PCR system (Applied Biosystems). The reaction system contained 5 μl of 2 × SYBR Mix, 0.5 μl of each primer, 1 μl of 10 × cDNA, and 10 μl of ddH_2_O. Reaction conditions were as follows: 2 min pre-denaturation at 95 °C, denaturation for 10 s at 95 °C, annealing for 30 s at 60 °C, 40 cycles. Relative expression of Bcl-2 and caspase-3 mRNA with GAPDH as the housekeeping gene to be used based on the 2-^ΔΔCT^ method was calculated. The primer sequences are as follows: Bcl-2, 5′-GGTGAACTGGGGGAGGATTG-3′, and 5′-AGAGCGATGTTGTCCACCAG-3'; caspase-3, 5′-GGAGCTTGGAACGCGAAGAA-3′, and 5′-ACACAAGCCCATTTCAGGGT-3'; and GAPDH, 5′-GGTGAACTGGGGGAGGGAGGATTG-3′, and 5′-ATGGTGGTGAAGACGCCAGTA-3'.

### 2.14 Chromatin immunoprecipitation (ChIP) assay

ChIP assays were performed to identify the histone modifications within the promotion regimes of caspase-3 and Bcl-2. The ischemic hemispheric brain tissue (150 mg) was measured according to the manufacturer's instructions. Briefly, protein-DNA cross-linking was first performed with 37% formaldehyde, followed by nuclear preparation, chromatin fragmentation, and ultrasonic fragmentation to obtain the protein-DNA cross-linking complexes. Chromatin was immunoprecipitated using the corresponding antibodies (H3K9ace rabbit Ab, Cell Signaling Technology, cat #9649; H3K27ace rabbit Ab, Cell Signaling Technology, cat #4353) and Protein G magnetic beads. The chromatin was then uncrosslinked, and the DNA was purified. Finally, the target gene bound to H3K9ace/H3K27ace was amplified using qPCR. The primer sequences used were as follows: Caspase-3 -502 bp ∼ −710 bp: 5′-CACACGCAGCGAATGGTAGA-3' (forward) and 5′-TCCGACTGCTTTGGGTCCTA-3' (reverse); Bcl-2 -286 bp ∼ −436 bp: 5′-AAGAGGATTCTGGTCCCCGT-3' (forward) and 5′-GATCGGTATCCACCAGACCG-3' (reverse).

The relative enrichment of H3K9ace/H3K27ace in the promoter region of the target genes in each group was compared as a percentage, that is, percent input = 2% x2^[c(T)2%input Sample-c(T)IP sample]^.

### 2.15 Statistical analysis

Materials were subjected using a one-way analysis of variance (ANOVA), indicated by the mean ± SD, performed by Prism 9.0, and a one-way ANOVA for comparison between the groups was performed. *P* < 0.05. meaningful.

## 3 Results

### 3.1 EA treatment attenuates I/R injury in the brain of MCAO rats

**Fig. 2:**
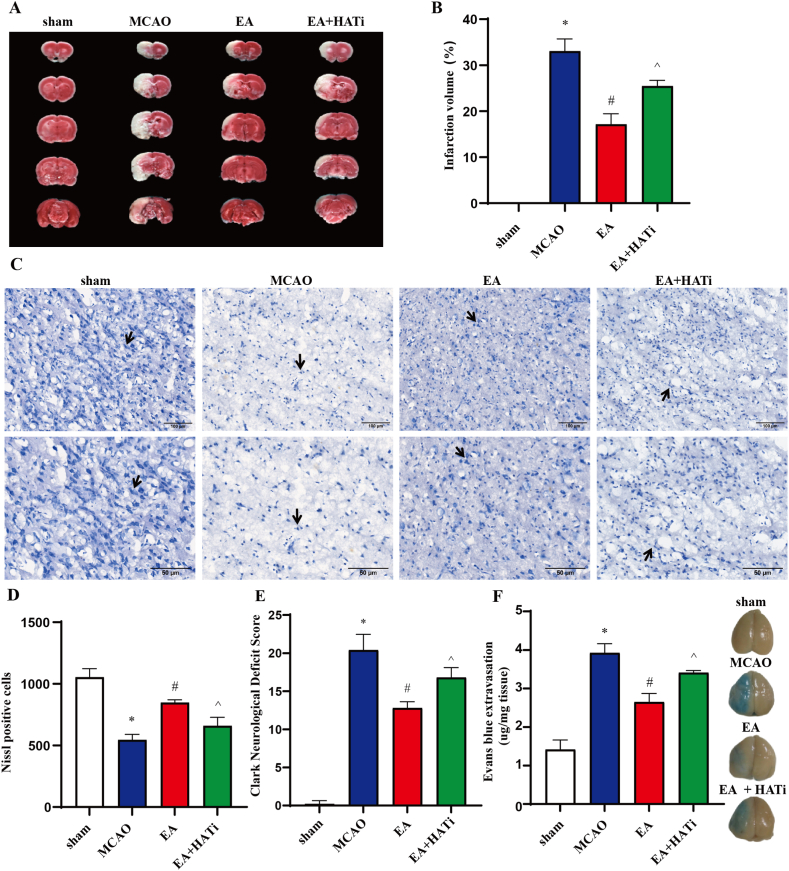
EA treatment attenuates I/R injury in the brain of MCAO rats. (**A**) TTC staining. (**B**) volume of cerebral infarction (n = 6). (**C**) Nissl staining. (**D**) number of Nissl bodies (n = 6). (**E**) Clark's Neurological Function Score (n = 5). (**F**) Evans blue extravasation (n = 5). The values are the mean ± standard deviation. *P < 0.05 vs. the sham group, ^#^*P* < 0.05 vs. the MCAO group, ^*P* < 0.05 vs. the EA group.

To assess the effect of EA on poststroke cerebral ischemic injury in rats, we examined the cerebral infarct volume, behavioral scores, neuronal damage, and BBB integrity. At 24 h post-ischemic stroke, the MCAO group exhibited significantly higher cerebral infarct volume and Clark neurological function scores than did the sham surgery group. However, EA treatment significantly reduced Clark scores and the percentage of cerebral infarct volume in the MCAO animals (*P* < 0.05) (Fig. 2A, B, E). Simultaneously, the MCAO group exhibited a sparse and lost arrangement of Nissl bodies, whereas EA treatment significantly increased the number of Nissl bodies (Fig. 2C and D). EB extravasation was significantly increased due to BBB damage after ischemia, whereas EA significantly reduced EB extravasation in MCAO animals (*P* < 0.05) (Fig. 2F). The EA + HATi group exhibited a significantly higher percentage of cerebral infarct volume and neurological function scores and increased rates of neuronal damage and EB extravasation compared to the EA group. However, these parameters were not as favorable as those observed in the EA group. It has been suggested that EA can effectively improve cerebral I/R injury and that acetylation inhibition can partially block the ameliorative effect of EA on pathological injury in stroke rats.

### 3.2 EA inhibits apoptosis in ischemic brain tissue of stroke rats

**Fig. 3:**
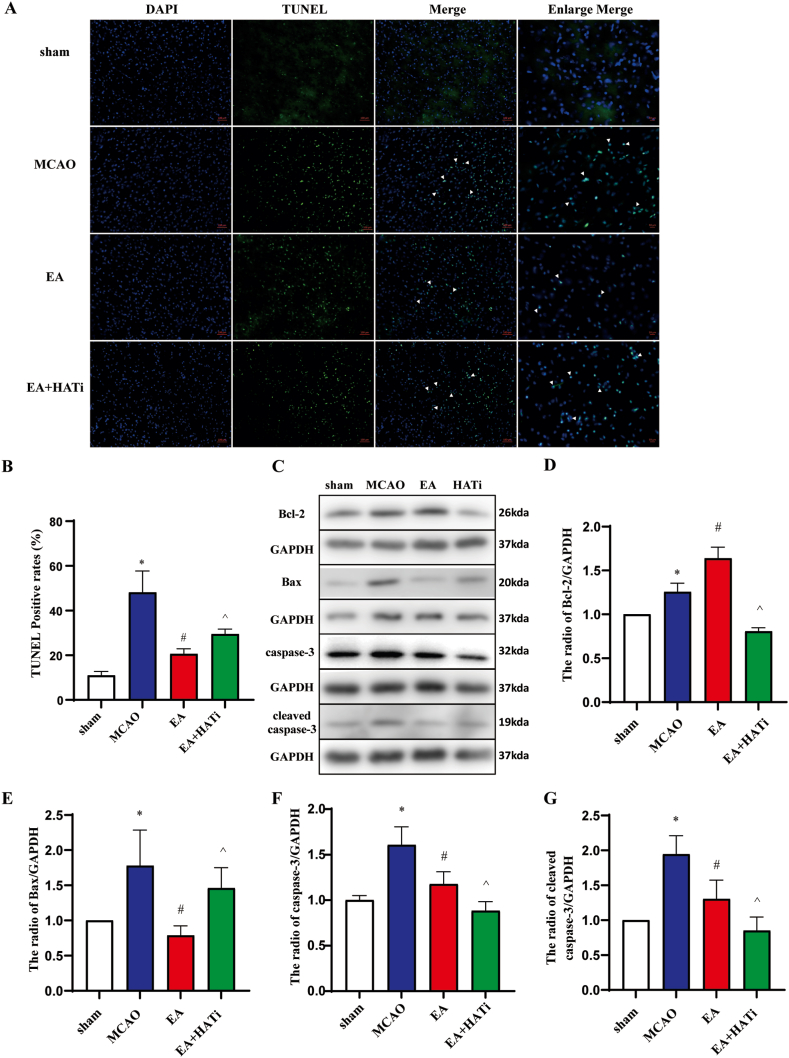
Electroacupuncture inhibits apoptosis in ischemic brain tissue of stroke rats. (**A** and **B**) TUNEL staining was performed to detect neuronal apoptosis in the ischemic cortex of mice (n = 6). (**C**) The protein expression levels of apoptotic proteins Bcl-2, Bax, caspase-3, and cleaved caspase-3 were assessed by performing Western blot. For original immunoblot images, please see Supplementary material 1. (**D**) Expression level of the apoptotic protein Bcl-2 in each treatment group (n = 4). (**E**) Expression level of the apoptotic protein Bax in each treatment group (n = 4). (**F**) Expression level of the apoptotic protein caspase-3 in each treatment group (n = 4). (**G**) Expression level of the apoptotic protein cleaved caspase-3 in each treatment group (n = 4). The information is presented as mean standard deviation (SD). The error bars represent standard deviation, and the statistical symbol indicates a statistical difference between the indicated columns, based on one-way ANOVA. *P < 0.05 vs. the sham group, ^#^*P* < 0.05 vs. the MCAO group, ^*P* < 0.05 vs. the EA group.

TUNEL-positive cells exhibited dense aggregation within the cell nuclei and displayed morphological characteristics of apoptosis. These cells were predominantly distributed in the ischemic cortex in the MCAO group. Compared with the MCAO group, the EA group showed a significant reduction in the percentage of TUNEL-positive cells. However, when comparing the EA + HATi group to the EA group, there was a significant increase in the percentage of TUNEL-positive cells (*P* < 0.05) (Fig. 3A and B).

Additionally, we measured the expression of apoptosis-related proteins, including Bcl-2, Bax, caspase-3, and cleaved caspase-3 in the ischemic hemisphere. These results indicate that I/R injury significantly increased the expression of Bcl-2, Bax, caspase-3, and cleaved caspase-3. EA treatment significantly enhanced the expression of Bcl-2 protein while reducing the expression of Bax, caspase-3, and cleaved caspase-3 proteins (*P* < 0.05) (Fig. 3C–G). Following treatment with the HATi inhibitor, compared with the EA group, the expression of Bcl-2, caspase-3, and cleaved caspase-3 proteins decreased, whereas the expression of Bax protein increased (*P* < 0.05) (Fig. 3C–G). These results suggest that EA can inhibit neuronal apoptosis in the ischemic cortex of rats with cerebral infarction, increase Bcl-2 protein expression, and decrease the expression of apoptosis-related proteins, such as Bax, caspase-3, and cleaved caspase-3.

### 3.3 EA treatment enhances histone acetylation in ischemic brain tissue of stroke rats

**Fig. 4:**
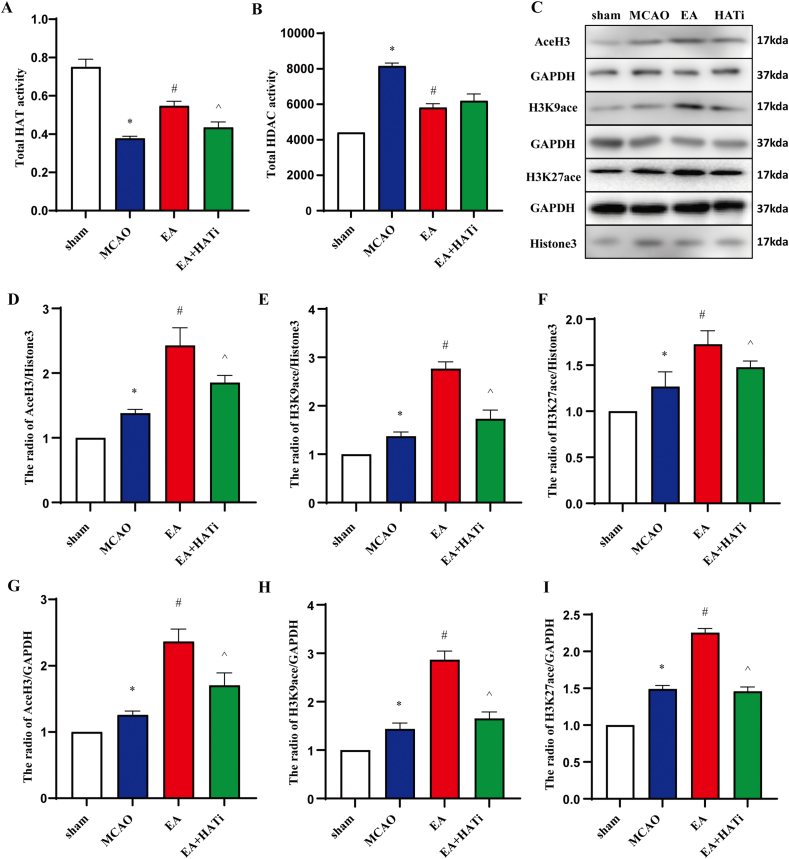
EA treatment enhances histone acetylation levels in the ischemic hemisphere 1 day after MCAO. The tissue was collected to isolate proteins for ELISA and Western blot. HAT and HDAC activity were measured by enzyme-linked immunosorbent assay (ELISA). (**A**) HAT activity (n = 5). (**B**) HDAC activity (n = 4). (**C–I**) Histone acetylation (H3, H3K9ace, and H3K27ace) levels were detected by Western blot and quantification analysis. Protein loading was normalized to Histone3 or GAPDH, which served as a control. For original immunoblot images, please see Supplementary material 2. The quantitation of blots was performed by densitometric analysis (n = 4). The information is presented as mean standard deviation (SD). The error bars represent standard deviation, and the statistical symbol indicates a statistical difference between the indicated columns, based on one-way ANOVA. *P < 0.05 vs. the sham group, ^#^*P* < 0.05 vs. the MCAO group, ^*P* < 0.05 vs. the EA group.

Histone acetylation is related to the acetylase activity of both HAT and HDAC. To observe the effect of EA on histone acetylation in the ischemic brain tissue of rats, changes in HAT and HDAC activity were examined after ischemic stroke. Twenty-four hours after I/R injury, HAT in the MCAO group exhibited distinctly less activity than did those in the sham group, in contrast to the EA treatment, resulting in increased activity of HAT. HAT activity was downregulated in the EA + HATi group compared to the EA group 24 h after I/R, indicating that HATi can partially block the regulation of HAT activity by EA (*P* < 0.05) (Fig. 4A). The results of the HDAC activity assay showed that HDAC activity in the MCAO group increased significantly by approximately 60%, and after EA treatment, HDAC activity in the EA group decreased by approximately 25% (*P* < 0.05) (Fig. 4B). The EA group was not significantly different from the EA + HATi group, indicating that HATi did not influence the effect of EA on HDAC activity.

Acetylation of Ace-H3, H3K9, and H3k27 is a well-studied histone modification that regulates the transcriptional activity of genes. Protein immunoblot analysis was performed to examine the effects of EA on histone proteins. The results indicated that the MCAO group had a higher expression of Ace-H3, H3K9ace, and H3K27ace 24 h after I/R than did the sham group. EA processing markedly enhanced the expression of Ace-H3, H3K9ace, and H3K27ace (*P* < 0.05). These findings indicate that EA increases the levels of Ace-H3, H3K9ace, and H3K27ace in brain tissue of the ischemic hemisphere after stroke. In addition, HATi partially inhibited the effects of EA (*P* < 0.05) (Fig. 4C–I). This indicated that the mechanism underlying the neuroprotective effects of EA may be associated with regulating gene transcription via H3K9ace and H3K27ace.

### 3.4 EA significantly enhanced the occupancy of H3K9ace and H3K27ace in the Bcl-2 gene promoter

**Fig. 5:**
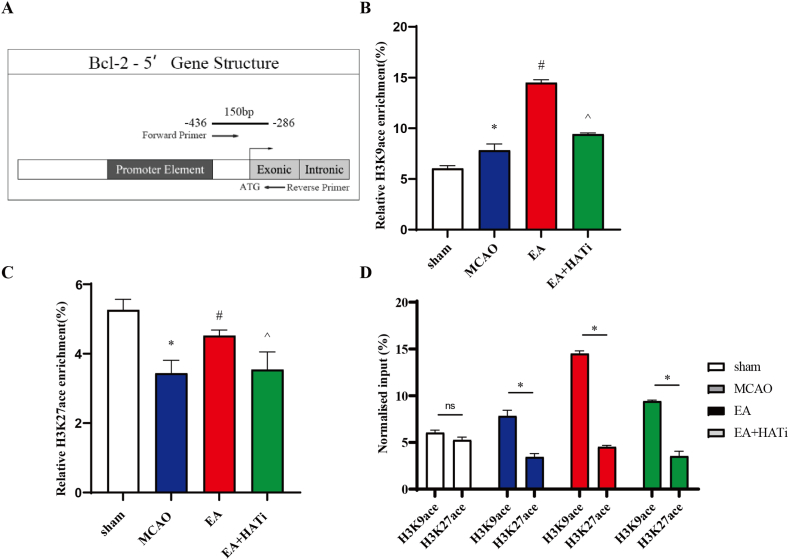
EA enhanced histone acetylation at the Bcl-2 gene promoters. (**A**) Schematic representation of the Bcl-2 gene promoter regions, relative position of the primers used for the ChIP experiment to the translational start (ATG). (**B** and **C**) Chromatin immunoprecipitation experiments using antibodies against global H3K9 and H3K27 acetylation were carried out on the Bcl-2 genes. Relative amounts of immunoprecipitated Bcl-2 promoter sequences compared to input chromatin were determined by real-time PCR (n = 3). (**D**) Comparison of H3K9ace and H3K27ace occupancy in the Bcl-2 gene promoter. The values are the mean ± standard deviation. *P < 0.05 vs. the sham group, ^#^*P* < 0.05 vs. the MCAO group, ^*P* < 0.05 vs. the EA group.

Next, we performed ChIP to investigate whether the modulatory effect of acupuncture on H3K9ace and H3K27ace proteins was related to Bcl-2 gene transcription activity. At the −286 bp-436 bp region of the H3K9ace locus of Bcl-2, our data showed increased occupation of the MCAO group with H3K9ace compared to that of the sham group. EA significantly enhanced the enrichment of H3K9ace compared to MCAO. The application of HATi reduced H3K9ace occupancy compared to the EA group (*P* < 0.05) (Fig. 5A and B). For the H3K27ace locus, H3K27ace occupancy in the MCAO group decreased compared to that in the sham group, whereas EA processing notably enhanced the enrichment of H3K27ace (*P* < 0.05). The application of HATi reduced H3K27ace occupancy compared to the EA group (Fig. 5A–C). Interestingly, we identified a higher H3K9ace occupied locus than the H3K27ace locus in all the groups (Fig. 5D).

### 3.5 EA significantly reduced the occupancy of H3K9ace and H3K27ace in the caspase-3 gene promoter

**Fig. 6:**
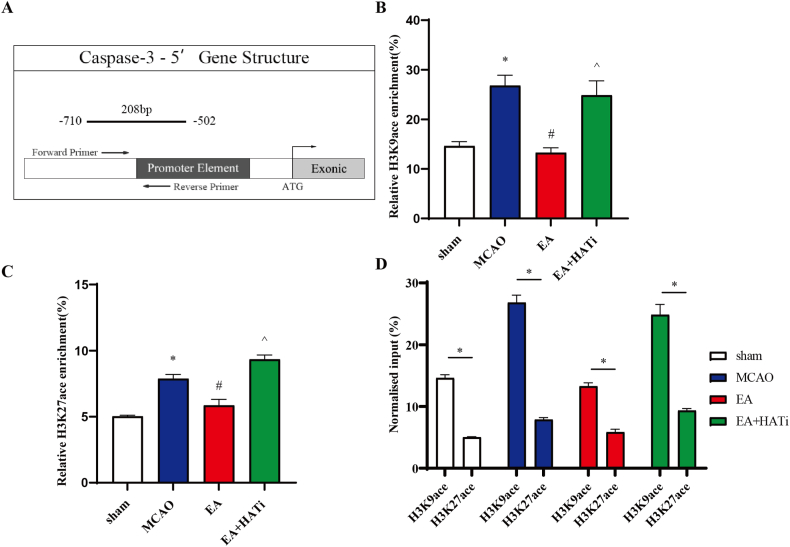
EA attenuates histone acetylation of caspase-3 gene promoter. (**A**) Schematic representation of the caspase-3 gene promoter region. (**B** and **C**) ChIP analyses were conducted on the caspase-3 promoter regions using anti-acetyl-histone H3K9 and anti-acetyl-histone H3K27. Enrichment was determined relative to input controls (n = 3). (**D**) The occupancy of H3K9ace in the gene promoter was significantly stronger than that of H3K27ace (n = 3). The values are the mean ± standard deviation. *P < 0.05 vs. the sham group, ^#^*P* < 0.05 vs. the MCAO group, ^*P* < 0.05 vs. the EA group.

In the −502 to −710 bp region of caspase-3, we performed amplification of the H3K9ace-DNA fragment and the H3K27ace-DNA fragment. The results of the H3K9ace-DNA fragment amplification revealed that the MCAO group had significantly larger H3K9 acetylation horizontal than did the sham group (*P* < 0.05), and the percentage of H3K9ace-enriched caspase-3 DNA fragments was almost twice as high as that of the sham group. However, EA treatment significantly reduced the H3K9ace occupancy. The application of HATi partially enhanced H3K9 acetylation (*P* < 0.05) (Fig. 6A and B). In parallel, the amplification fragment of the H3K27ace-DNA complex showed that the MCAO group had greater occupancy of H3K27ace than did the sham group, whereas EA treatment significantly downregulated H3K27ace enrichment. Combined with HATi intervention, the effect of EA was reversed (*P* < 0.05) (Fig. 6A–C). In addition, the occupancy by H3K9ace at the gene promoter for the caspase-3 gene was significantly stronger than that of H3K27ace (Fig. 6D).

### 3.6 EA promotes Bcl-2 mRNA expression and inhibits caspase-3 mRNA expression

**Fig. 7:**
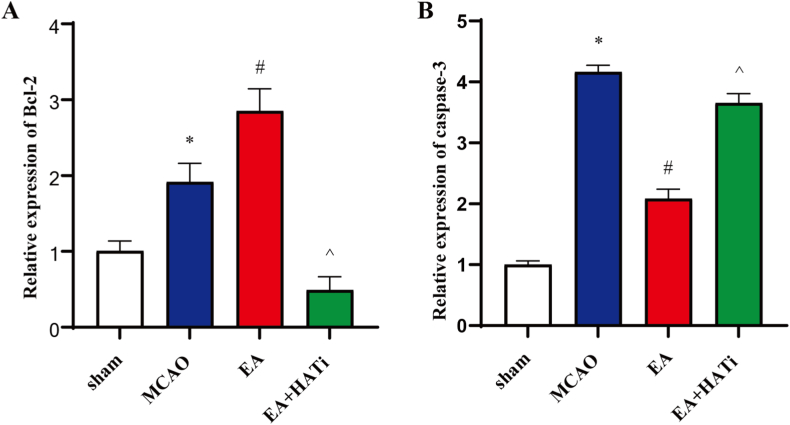
Quantitative real-time PCR analysis confirms the results of ChIP-PCR. Enrichment of promoter regions of Bcl-2 and caspase-3 was confirmed by real-time PCR. (**A**) Expression of Bcl-2 mRNA (n = 3). (**B**) Expression of caspase-3 mRNA (n = 3). Values are the mean ± standard deviation; error bars represent SD. *P < 0.05 vs. the sham group, ^#^*P* < 0.05 vs. the MCAO group, ^*P* < 0.05 vs. the EA group.

To confirm the above ChIP results, we examined the mRNA expression levels of Bcl-2 and caspase-3 in the ischemic hemisphere. The results showed that I/R injury significantly increased the mRNA expression of Bcl-2 and caspase-3 (*P* < 0.05). EA treatment markedly increased Bcl-2 mRNA expression; however, the application of HATi significantly decreased its expression (Fig. 7A). In contrast, EA treatment decreased caspase-3 mRNA expression, whereas the use of HATi reversed this effect (*P* < 0.05) (Fig. 7B).

## 4 Discussion

EA has been widely used to treat stroke owing to its simplicity, convenience, effectiveness, and low cost. Extensive studies have demonstrated that EA has a significant positive outcome in ameliorating neurological disorders after stroke [20,34]. Preclinical experimental studies have shown that EA can inhibit inflammation [35], protect antioxidant enzymes [36], improve neuronal cell plasticity [37], inhibit apoptosis [23], and reduce brain damage after ischemic stroke. The Baihui acupoint (GV20) is a routine acupoint for the EA treatment of stroke. Several experimental studies have reported that EA treatment at the GV20 acupoint has neuroprotective effects in rats with MCAO [20,38,39]. Our previous studies have also shown that EA can effectively alleviate the I/R injury by reducing the inflammatory response in the ischemic brain and restoring the balance between Tregs and γδ T cells [32,40]. Therefore, in this study, the GV20 acupoint was selected to treat ischemic stroke in rats. Our results demonstrated that EA treatment significantly reduced cerebral infarct volume, BBB permeability, and neuronal damage and improved neurological function in ischemic stroke rats, per earlier findings [19,38]. However, the mechanism underlying the protective effects of EA on the brain requires further investigation.

It is well established that apoptosis is closely related to the loss of hypoxic-ischemic neurons in the nervous system [24,41], the balance that exists between pro-apoptotic proteins (caspase-3) and resistant proteins (Bcl-2) determines whether a neuron survives or dies [[42], [43], [44], [45], [46]]. After ischemic stroke, pathological brain damage is aggravated by increased apoptosis, which manifests as a sharp increase in caspase-3 and a slight upregulation of Bcl-2 levels. Nakamura et al. [47] demonstrated that the overexpression of Bcl-2 in transgenic mice or via viral vectors reduced the number of reactive astrocytes in the ipsilateral dorsal and ventral thalamus, thus protecting the brain from damage. Chen et al. [48] found that endogenous knockout of antisense Bcl-2 aggravates ischemic damage in the brains of rats. Experimental studies have demonstrated that caspase-3 activity facilitates an intense delay in neuronal mortality in response to short-lived ischemia. Chen et al. found that the ventricular infusion of caspase-3 inhibitors reduced caspase-3 levels in the hippocampal area, dramatically diminished cell mortality in the CA1 region, and mitigated DNA breakdown after 7 days of ischemia [49]. Similarly, Schielke et al. demonstrated that knocking out the caspase-3 gene reduced cerebral oedema and improved brain injury [50]. In this study, a notable reduction in neuronal apoptosis was observed within the ischemic cortex of rats with cerebral infarction following EA treatment, consistent with Ding et al.'s findings [51]. In addition, we also found that the expression of the proteins Bcl-2, Bax, caspase-3, and cleaved caspase-3 was elevated following a stroke event; EA downregulated the expression of Bax, caspase-3, and cleaved caspase-3 protein while upregulating Bcl-2 protein expression in the ischemic brain of stroke rats. This suggests that EA alleviates ischemic brain injury by reducing neuronal cell apoptosis, consistent with previous experiments' results [[11], [12], [13],^36^,^52^,^53^]. However, the mechanism by which EA regulates the expression of Bcl-2 and caspase-3 remains poorly understood.

Histone lysine acetylation marks the open chromatin in epigenetic alterations and facilitates active gene expression. Their dynamic conditioning is achieved through the cooperative action of HAT and HDAC [54]. HAT are a class of enzymes involved in regulating chromatin structure and gene expression. They catalyze the acetylation of core histones by adding acetyl groups from acetyl coenzyme A to the lysine residues of histones [55]. In contrast, HDAC regulate deacetylation by hydrolyzing the acetyl portion of lysine residue [56]. In a previous study, we found that EA could mediate the expression of several HDAC in the ischemic brains of rats following stroke [28]. This suggests that the neuroprotective effects of EA in stroke rats may be related to histone acetylation. In this study, we confirmed that HAT enzyme activity decreased and HDAC enzyme activity increased in the cerebral ischemic tissue of MCAO rats, whereas EA treatment upregulated HAT enzyme activity and downregulated HDAC enzyme activity. Simultaneously, the modulatory effect of EA was partially prevented by the HATi application. Histone 3 (H3) is one of the most extensively studied proteins involved in histone modification of histones [25]. Acetylation of histone H3 mainly occurs at lysine 9 (H3K9) and 27 (H3K27). Acetylated H3K9 is related to active transcription [57], and acetylated H3K27 is a recognized marker of active enhancers [58,59]. Xu et al. detected an association between the acetylation of H3K9 at the GATA4/Nkx2.5 promoter region and the expression of both genes and suggested that Gcn5 is a pivotal HAT that regulates H3K9 acetylation at the GATA4/Nkx2.5 promoter region [60]. Stief et al. suggested that ENT1 export is regulated by H3K27 acetylation, whereas H3K27 demethylase is not required for ENT1 expression [61]. We found that EA treatment significantly enhanced the acetylation of H3, H3K9, and H3K27. Similar to the enzyme activity, the effects of EA were diminished by HATi. ChIP assay results indicated that EA significantly enhanced the occupancy of H3K9ace and H3K27ace in the Bcl-2 gene promoter and reduced the occupancy of H3K9ace and H3K27ace in the caspase-3 gene promoter. Moreover, the regulatory role of EA on histone acetylation in the promoter regions of Bcl-2 and caspase-3 was reversed by HATi. Subsequently, we found that HATi could reverse the upregulation of Bcl-2 gene expression and the downregulation of caspase-3 gene expression induced by EA at the transcriptional level. This was consistent with the changes in the enrichment of H3K9ace and H3K27ace in the transcriptional initiation regions of these two genes, indicating that EA regulates the transcriptional activities of Bcl-2 and caspase-3 through histone acetylation. In addition, the neuroprotective effects of EA and its inhibitory effect on apoptosis were partially reversed by HATi. The neuroprotective mechanism of EA in inhibiting apoptosis after cerebral ischemic stroke may be related to histone acetylation.

## 5 Conclusion

**Fig. 8:**
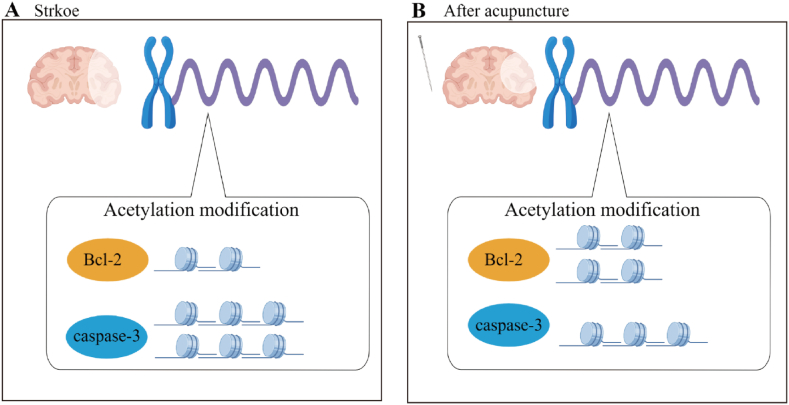
Possible mechanistic scenarios for the cerebral protective effect of EA through histone acetylation modifications.

Collectively, our data demonstrate that EA could mediate the expression levels of Bcl-2 and caspase-3 genes in MCAO rats, by regulating the occupancy of acetylated H3K9/H3K27 at the promoters of these two genes (Fig. 8A and B), thus exerting a cerebral protective effect in ischemic injury. These findings offer new insight into the underlying neuroprotective mechanisms of acupuncture, which may have clinical implications for stroke treatment.

## Data availability statement

All the data used to support the findings of this study are included within the article and the supplementary materials. Additional data related to this paper may be requested from the authors.

## Consent for publication

All authors have read and approved the manuscript.

## Ethics declarations

This study was reviewed and approved by [Ethics Committee for Animal Experiments of Nanjing University of Traditional Chinese Medicine], with the approval number: [201911A018].

## CRediT authorship contribution statement

**Lingling Meng:** Writing – original draft, Validation, Methodology, Data curation. **Bufan Wu:** Writing – review & editing, Validation, Methodology. **Ling OuYang:** Validation, Methodology. **Rou Peng:** Validation, Methodology. **Yonglin Chen:** Validation, Methodology. **Zhijuan Tang:** Validation, Methodology. **Min Zhang:** Methodology. **Tanqing Xu:** Methodology. **Yaling Wang:** Methodology. **Shengfeng Lu:** Project administration, Methodology, Conceptualization. **Xinyue Jing:** Writing – review & editing, Project administration, Conceptualization. **Shuping Fu:** Writing – review & editing, Supervision, Project administration, Methodology, Data curation.

## Declaration of competing interest

The authors declare that they have no known competing financial interests or personal relationships that could have appeared to influence the work reported in this paper.
