# Supplementary material for: Electroacupuncture regulates histone acetylation of Bcl-2 and Caspase-3 genes to improve ischemic stroke injury
Source: Heliyon. 2024 Mar 4;10(6):e27045. doi: 10.1016/j.heliyon.2024.e27045 (PMC10945129; doi:10.1016/j.heliyon.2024.e27045)
Supplement: Multimedia component 1 [file mmc1.pptx]

## Slide 1
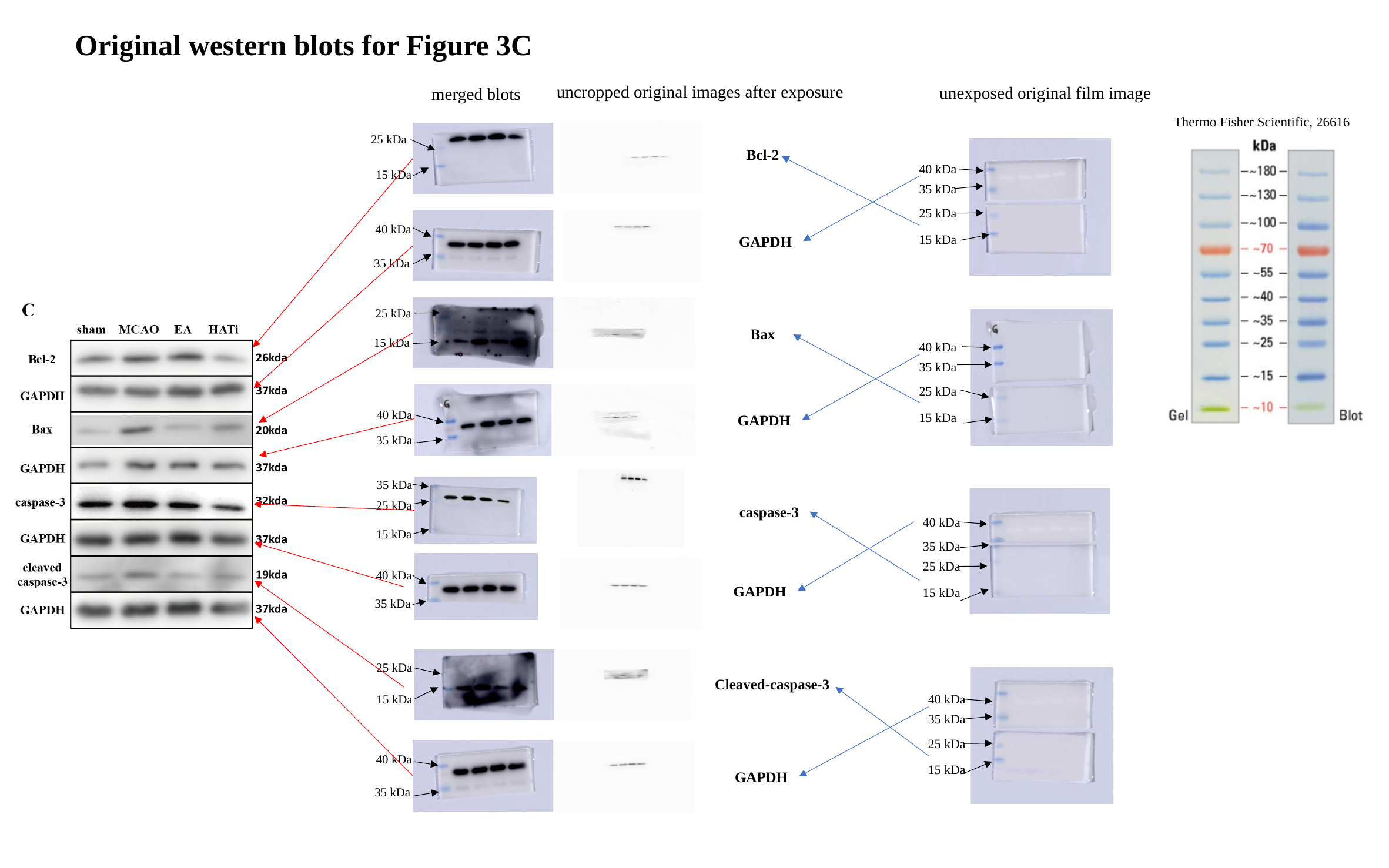

Original western blots for Figure 3C
uncropped original images after exposure
unexposed original film image
merged blots
Thermo Fisher Scientific, 26616
25 kDa
Bcl-2
40 kDa
15 kDa
35 kDa
25 kDa
40 kDa
15 kDa
GAPDH
35 kDa
25 kDa
Bax
15 kDa
40 kDa
35 kDa
25 kDa
40 kDa
15 kDa
GAPDH
35 kDa
35 kDa
25 kDa
caspase-3
40 kDa
15 kDa
35 kDa
25 kDa
40 kDa
GAPDH
15 kDa
35 kDa
25 kDa
Cleaved-caspase-3
40 kDa
15 kDa
35 kDa
25 kDa
40 kDa
15 kDa
GAPDH
35 kDa
