# Supplementary material for: Electroacupuncture regulates histone acetylation of Bcl-2 and Caspase-3 genes to improve ischemic stroke injury
Source: Heliyon. 2024 Mar 4;10(6):e27045. doi: 10.1016/j.heliyon.2024.e27045 (PMC10945129; doi:10.1016/j.heliyon.2024.e27045)
Supplement: Multimedia component 2 [file mmc2.pptx]

## Slide 1
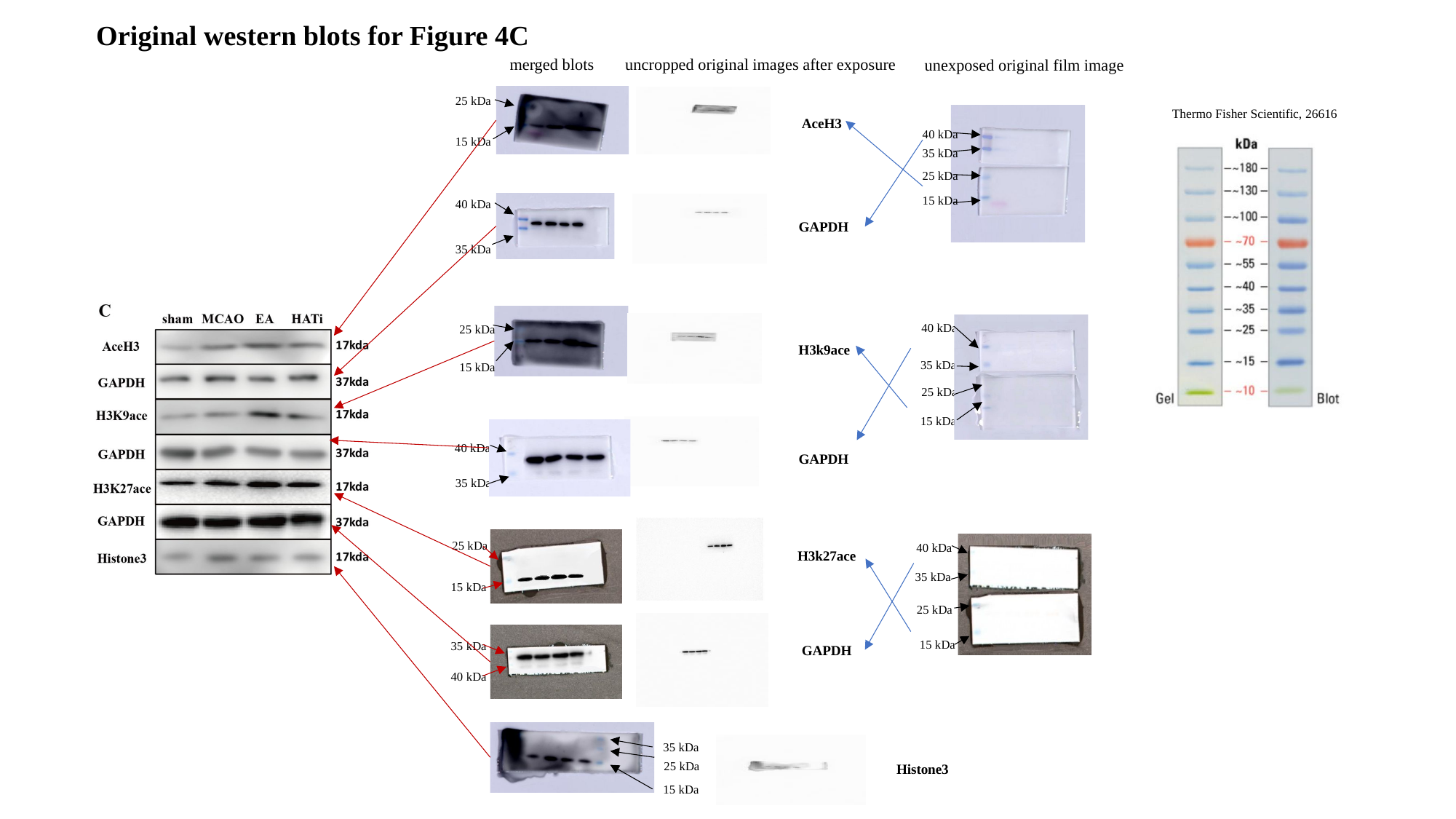

Original western blots for Figure 4C
uncropped original images after exposure
merged blots
unexposed original film image
25 kDa
Thermo Fisher Scientific, 26616
AceH3
40 kDa
15 kDa
35 kDa
25 kDa
15 kDa
40 kDa
GAPDH
35 kDa
40 kDa
25 kDa
H3k9ace
35 kDa
15 kDa
25 kDa
15 kDa
40 kDa
GAPDH
35 kDa
25 kDa
40 kDa
H3k27ace
35 kDa
15 kDa
25 kDa
15 kDa
35 kDa
GAPDH
40 kDa
35 kDa
25 kDa
Histone3
15 kDa
